# Electrophysiological differences in older and younger adults’ anaphoric but not cataphoric pronoun processing in the absence of age-related behavioural slowdown

**DOI:** 10.1038/s41598-020-75550-3

**Published:** 2020-11-06

**Authors:** Seçkin Arslan, Katerina Palasis, Fanny Meunier

**Affiliations:** 1 Université Côte d’Azur, CNRS, BCL, 24 Avenue des Diables Bleus, 06357 Nice Cedex 4, France; 2 Faculty of Arts, University of Groningen, Groningen, The Netherlands; 3 Present Address: Faculty of Arts, Neurolinguistics, Harmoniebuilding, PO Box 716, 9700 AS Groningen, The Netherlands

**Keywords:** Cognitive ageing, Language

## Abstract

This study reports on an event-related potentials experiment to uncover whether per-millisecond electrophysiological brain activity and analogous behavioural responses are age-sensitive when comprehending anaphoric (referent-first) and cataphoric (pronoun-first) pronouns. Two groups of French speakers were recruited (young *n* = 18; aged 19–35 and older adults *n* = 15; aged 57–88) to read sentences where the anaphoric/cataphoric pronouns and their potential referents either matched or mismatched in gender. Our findings indicate that (1) the older adults were not less accurate or slower in their behavioural responses to the mismatches than the younger adults, (2) both anaphoric and cataphoric conditions evoked a central/parietally distributed P600 component with similar timing and amplitude in both the groups. Importantly, mean amplitudes of the P600 effect were modulated by verbal short-term memory span in the older adults but not in the younger adults, (3) nevertheless, the older but not the younger adults displayed an additional anterior negativity emerging on the frontal regions in response to the anaphoric mismatches. These results suggest that pronoun processing is resilient in healthy ageing individuals, but that functional recruitment of additional brain regions, evidenced with the anterior negativity, compensates for increased processing demands in the older adults’ anaphora processing.

## Introduction

*Does syntactic processing decline with age?* This question has been examined across different languages using a variety of experimental methods; nevertheless, research has not been able to provide a confident answer yet. A number of studies have reported that older adults perform more slowly and less accurately than younger adults when responding to sentence judgement tasks^1–4^ including tasks that require pronoun interpretation^5–7^. Some accounts^8,9^ predict a general cognitive slow-down in the old age, whereas others suggest that any potential decline in syntactic processing may not be unitary. Particularly, Kemtes and Kemper^10^ reported that age-related changes in syntactic processing are modulated by reading span–a working memory (WM) measure that requires reading sentences with increasing length and complexity^11^. Several studies have then indicated that older adults perform poorly in tasks that require WM-intensive, complex syntactic processing^1,12–15^. By contrast, there is also evidence that syntactic processing is resilient to ageing effects^16–21^. In an fMRI study, Tyler and colleagues^16^ administered a word-monitoring task while groups of young and older adults listened to anomalous spoken sentences. Their findings indicated that bilateral superior temporal gyri and left inferior frontal gyrus (IFG) were similarly activated in both groups; however, older adults with diminished grey matter density in the left IFG showed greater brain activity in the right hemisphere regions homologous to the left IFG. Further studies have also adduced evidence for functional recruitment of additional neural regions compensating for the reduced brain activity in the sentence processing network in older adults^22,23^. Furthermore, Campbell et al.^19^ showed that older adults performed indifferently from younger adults in responding to spoken sentence comprehension tasks, processing of which similarly recruited the frontotemporal network in both groups. Taken together, these findings suggest that syntactic processing is maintained across adulthood despite naturally incurring neurobiological changes in the brain. Critically, an unclarity in our current understanding is to which extent the maintenance of syntactic processing is dependent on interpretive processes during real-time sentence comprehension. In both the accounts presented here (i.e., ‘syntactic decline’ vs. ‘syntactic resilience’), studies extensively used experimental designs where participants read or listened to sentence stimuli to make post-interpretive judgements, leaving the precise online time-course of syntactic processing less explored. In fact, a number of studies using word-by-word listening tasks suggest that online syntactic processing is maintained in older adults despite low performance in offline sentence judgements^24^; nevertheless, this technique does not investigate the temporal dynamics of brain activity during sentence processing. Hence, using an event-related potentials design in EEG, the current study aimed to examine per-millisecond electrophysiological brain activity affiliated with anaphoric (referent-first) and cataphoric (pronoun-first) pronoun processing in groups of young (aged 19–35) and older (aged 57–88) adult speakers of French.

Pronouns present a referential dependency relationship with another entity that they refer to (i.e., their ‘antecedent’). Anaphoric pronouns appear after their antecedents in the sentence or discourse (e.g., Professor Niçois_i_ fell off his chair, after *he*_i_ began to lecture), while cataphoric pronouns come before their antecedents in the context (e.g., After *he*_i_ began to lecture, Professor Niçois*_i/j_ fell off his chair). Resolving anaphoric pronouns has been proposed to depend on storing and retrieving potential antecedents from the memory during sentence processing, and the time course of this process has been rather well studied^25–27^. In contrast, working out the antecedent of a cataphoric pronoun has been assumed to trigger a *search mechanism*; that is, once a cataphoric pronoun has been encountered, an active search probes the upcoming sentence to find its antecedent^28,29^. A typical design used in pronoun processing research is the gender-mismatch paradigm, in which participants read sentences with pronouns that mismatch their antecedents in gender (e.g., Maria/*he). Studies using this paradigm have observed disrupted reading times and evoked P600 ERP components time-locked to the critical pronoun^27,30–34^. For instance, Osterhout and Mobley^32^ used sentences with reflexive pronouns mismatching their potential antecedents in gender (e.g., ‘The successful *woman* congratulated herself/*himself on the promotion’) and reported that the sentence materials with mismatching pronouns elicited a P600 component. The presence of P600 effects in gender-mismatch conditions is argued to reflect re-analysis and recovery from the syntactic anomaly^35–38^, as well as incremental processes associated with referential processing^39,40^.

Studies on cataphoric pronouns are comparatively rarer and only few directly compare anaphoric and cataphoric pronoun processing^41,42^. Kennison et al.^41^ used a self-paced reading paradigm with anaphoric and cataphoric pronouns and reported that both anaphoric and cataphoric gender-mismatches led to reading disruptions at the critical pronoun/antecedent regions, but that longer word-by-word reading times emerged in the anaphoric pronoun conditions. In an ERP study, Pablos et al.^29^ found that gender-mismatches in sentences with structurally unconstrained cataphoric co-reference (e.g., ‘Her assistants found out that *Lodewijk_*masc*_… but Mirjam_fem_’) elicit an anterior negativity effect time-locked to the first potential antecedent, while no effects were observed in the constrained conditions (i.e., when coreference with the first potential antecedent is ruled out for structural reasons). Pablos et al.^29^ argued that the anterior negativity reflects a failure to find an appropriate antecedent during cataphoric pronoun processing.

In older adults, pronoun processing has been shown to be prone to memory constraints^5,43^. Data from an eye-tracking-during-reading study^44^ showed that both young and older adults had increased reading times on mismatching reflexive pronouns, yet older adults compensated for this decline when additional sentential context was provided. A study^7^ on a life-span sample of adults (aged 18–81) reported data from self-paced reading experiments addressing interpretation of locally ambiguous reflexive pronouns in complex relative clauses. This study found that both WM span and print exposure predict how pronoun interpretation ability changes in the course of adulthood. There are only a handful of ERP studies on pronoun/syntactic processing in older adults, however, the available research on more broad language-related ERP effects has shown that older adults’ ERP components, particularly N400 effects in lexical processing, are delayed and reduced in amplitude (see Wlotko, Lee and Federmeier^45^ for reviews). Furthermore, oscillatory dynamics of syntax-related brain potentials are found to be sensitive to ageing^46^. Of particular relevance, Kemmer and colleagues^47^ examined pronoun processing in a group of older adults using a gender-mismatch design (e.g., ‘The grateful niece asked herself/*themselves…’). The authors found that mismatching pronouns yielded a P600 effect without any differences between old and younger adults, although the older adults tended to perform less accurately and more slowly in their behavioural responses to mismatch sentences. Interestingly, Kemmer and her colleagues reported significant group effects for the distribution of the P600 effect: in older adults, the observed positive brain potentials were laterally symmetrical, while in younger adults the effect was larger over posterior sites and the left hemisphere. Alatorre-Cruz et al.^48^ critically examined impact of WM-load on older adults’ processing of gender/number agreement conditions. The authors reported that when the sentence material had a high WM-load (i.e., was structurally complex), older adults showed smaller amplitudes in the P600 component compared to the low WM-load condition, while there were no group differences for low and high WM-load sentences.

Summarising, older adults’ pronoun processing is affiliated with slower reading/response times compared to younger adults (e.g.^5,43^), whilst studies reporting time-sensitive neurophysiological measurements either indicate no group differences in mean ERP amplitudes^47^ or point to significant individual differences in modulation with the WM load^7,48^. Therefore, the state-of-the-art cannot provide a confident answer to whether and to which extent syntactic processing during pronoun comprehension declines in older adults. A fundamental issue here, also discussed by Peelle^49^, appears to be distinctions between interpretive (i.e., online activity pertaining to per-millisecond time course) and post-interpretive processes, see also^46^. The latter often represent participants’ latency and correctness rates in responses to end-of-sentence judgement tasks, which require enhanced cognitive resources rather than syntactic processing per se. Tasks that rely on such behavioural responses only may be problematic because observed age-related decline effects could in fact be related to a rather domain-general cognitive decline; see^23,50^ for discussions. Furthermore, research is largely biased to English (and/or Germanic languages), leaving aside languages where pronouns are phonological clitics (i.e., items lacking independent stress and hence obligatorily attaching to a stressed element). It is unknown whether such cross-linguistic differences influence age-related pronoun processing.

To address these gaps, the current study examines processing of anaphoric and cataphoric pronouns during reading comprehension in an ERP/EEG study with young (aged 19–35, *mean* = 22.05) and older (aged 57–88, *mean* = 64.73) groups of adult speakers of French – a language where subject pronouns are clitics (see Method). ERPs allow us to examine the precise time-course of pronoun processing during online sentence comprehension and the temporal dynamics of its affiliated electrophysiological brain activity. Our study has two major aims. The first aim is to unveil whether anaphoric and cataphoric pronoun processing is maintained in older adults with regard to per-millisecond electrophysiological brain activity. If syntactic processing is resilient to naturally incurring neurobiological changes in the ageing process, then we should find no group differences in old and younger adults’ behavioural responses, despite electrophysiological differences. Alternatively, if ageing specifically impacts post-interpretable processes, we should find no group differences in ERPs time-locked to the critical pronouns but find a decline in older adults’ end-of-sentence responses. Furthermore, we explore whether any behavioural/P600 differences are modulated by memory constraints by employing an independent measurement of verbal short-term memory. Second, we aim to uncover whether there are any processing asymmetries between anaphoric and cataphoric pronouns in relation with young and older adults’ behavioural and ERP responses. The rationale here is that if anaphoric and cataphoric pronoun resolution rely on different cognitive processes (i.e., storing and retrieval of antecedents in the memory for anaphors vs. antecedent-search mechanisms for cataphors^28,29,41,42^), we should be able to observe critical condition differences in behavioural and/or ERP effects. Following the available behavioural findings^41^ we expect anaphoric antecedent-pronoun mismatches to evoke larger effects (i.e., greater amplitude of ERP effects) than cataphoric pronoun-antecedent mismatches.

## Results

### Behavioural results

Table 1 reports the accuracy and response times (RTs) data from behavioural responses, and the outputs from (generalized) mixed-effects regression models computed on these data. The older adults showed slightly higher accuracy estimates than the younger adults (85% vs. 81%) with a significant interaction of Group × Condition. This interaction with Group is largely modulated by the fact that the older adults performed more accurately than the younger adults in responding to the cataphoric pronoun conditions (86% vs. 78%; ß = 1.01, SE = 0.35, z = 3.082, *p* = 0.002) but not to the anaphoric pronoun conditions (85% vs. 83%; ß = 0.43, SE = 0.34, *z* = 1.25, *p* = 0.21), as confirmed by post-hoc Tukey tests. Importantly, the fixed-effect of verbal short-term memory (vSTM) and a three-way interaction of Group × Mismatch × vSTM proved significant, evidencing that the participants’ accuracy in responding to gender mismatches was modulated by verbal memory span. This means that older adults with a reduced verbal memory span performed more poorly in detecting gender-violations than those with a higher memory span (ß = 0.89, SE = 0.17, z = 5.17, *p* < 0.001). This difference was rather less pronounced in the younger adults (ß = 0.25, SE = 0.08, z = 2.96, *p* = 0.003). Regarding the RTs data, the model outputs showed significant fixed-effects of Mismatch and Condition but not of Group. This evidenced that average RTs to match conditions were longer than to mismatch ones (2151 ms vs. 1786 ms), and that RTs to cataphoric pronoun conditions were longer than their anaphor counterparts (2091 ms vs. 1853 ms) without being moderated by any group differences. A significant interaction of Group × Mismatch indicates that the older adults responded to the match conditions with longer RTs than the younger adults (2351 ms vs. 1975 ms; ß = 0.34, SE = 0.15, z = 2.21, *p* = 0.02), while group differences in the mismatch conditions were not statistically significant (1999 ms vs. 1599 ms; ß = 0.22, SE = 0.15, z = 1.34, *p* = 0.15). No further critical differences were observed.

**Table 1:** Mean accuracy and response times in milliseconds per condition per group, and statistical outputs from (generalized) mixed-effects linear regression performed on behavioural data.

Descriptive mean statistics	Accuracy				Response times			
	Young adults		Older adults		Young adults		Older adults	
Anaphor match mean (SD)	0.85 (0.37)		0.85 (0.35)		1929.34 (2220.03)		2268.00 (1915.18)	
Anaphor mismatch mean (SD)	0.83 (0.37)		0.84 (0.34)		1395.49 (1474.87)		1833.34 (1853.70)	
Cataphor match mean (SD)	0.80 (0.39)		0.87 (0.33)		2022.86 (2168.02)		2437.68 (1945.28)	
Cataphor mismatch mean (SD)	0.77 (0.42)		0.86 (0.34)		1795.23 (2141.29)		2159.33 (5197.37)	

Model outputs	ß	SE	z-value	*p*	ß	SE	t-value	*p*
Intercept	**2.00**	**0.17**	**11.59**	** < 0.001**	**7.19**	**0.08**	**86.95**	** < 0.001**
**Fixed-effects**								
Group	** − 0.76**	**0.33**	** − 2.31**	**0.02**	− 0.20	0.16	− 1.25	0.21
Mismatch	0.06	0.12	0.53	0.59	** − 0.31**	**0.02**	** − 10.84**	** < 0.001**
Condition	− 0.03	0.11	− 0.29	0.76	**0.10**	**0.02**	**3.65**	** < 0.001**
vSTM	**0.44**	**0.16**	**2.65**	**0.008**	− 0.09	0.08	− 1.17	0.25
**Two-way interactions**								
Group × mismatch	− 0.33	0.23	− 1.43	0.16	**0.16**	**0.05**	**2.77**	**0.005**
Group × condition	** − 0.66**	**0.23**	** − 2.81**	**0.004**	0.05	0.05	0.97	0.33
Mismatch × condition	− 0.13	0.24	− 0.56	0.56	0.06	0.05	1.10	0.27
Group × vSTM	− 0.21	0.33	− 0.64	0.53	0.05	0.16	0.29	0.77
Mismatch × vSTM	0.22	0.12	1.76	0.07	** − 0.06**	**0.02**	** − 2.29**	**0.02**
Config × vSTM	0.21	0.12	1.66	0.09	− 0.02	0.02	− 0.89	0.36
**Three-way interactions**								
Group × mismatch × condition	− 0.21	0.47	− 0.45	0.65	0.13	0.11	1.16	0.24
Group × mismatch × vSTM	** − 0.77**	**0.25**	** − 3.07**	**0.002**	0.01	0.05	0.23	0.81
Group × condition × vSTM	− 0.20	0.24	− 0.82	0.41	0.01	0.05	0.19	0.84
Mismatch × condition × vSTM	0.22	0.25	0.89	0.37	0.06	0.05	1.14	0.25
**Four-way interactions**								
Group × mismatch × condition × vSTM	0.54	0.49	1.10	0.27	0.03	0.11	0.32	0.74

Estimates (*ß*) for logit accuracy and log-transformed response times.

SD = Standard deviation, SE = Standard error, *p*-values in the linear models were calculated with the Satterthwaite's approximation.

*p* values < 0.05 are bolded.

### ERP results

**Figure 1:**
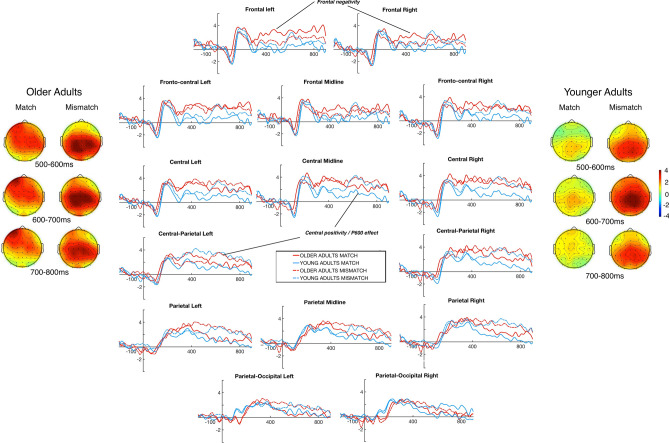
Grand averaged ERPs for *anaphoric pronoun match and mismatch conditions* across the fifteen regions of interest, frontal negativity and central positivity components are indicated in the plots. Dashed red lines represent mismatch conditions and solid lines represent match conditions. Topographic maps show distribution of averaged ERPs between 500 and 800 ms. Positive μV potential values are plotted up in the x-axes and time in millisecond is plotted in the y-axes. The plots were generated using the EEGLAB software toolbox.

An initial visual inspection showed that in both the groups (young vs. older) and conditions (anaphors vs. cataphors), a N170 component was present (followed by a positivity) in the 100–170 ms range, which is a typical ERP response to orthographic word recognition. Both conditions evoked large central/posterior positivity (see Figs. 1, 3). Table 2 displays outputs from a global ANOVA, which showed that between 300 and 500 ms, the anaphoric pronoun conditions evoked greater positive responses than the cataphoric pronoun conditions (2.19 μV vs. − 0.10 μV), and that the older adults’ responses were more positive than those of the younger adults (1.98 μV vs. 0.10 μV). However, given the nonsignificant main-effect of Mismatch and nonsignificant interactions between Mismatch and Group, greater positivity evoked in the older group cannot be affiliated with pronoun mismatches in this time-window. Between 500 and 700 ms, sentences with a mismatch evoked more positive responses (2.30 μV) than sentences without a mismatch (1.09 μV); sentences with anaphoric pronouns evoked more positive responses than those with cataphoric pronouns (2.04 μV vs. 1.36 μV). Although the main-effect of Group and interactions between Group and Mismatch were not statistically significant, we found a significant three-way interaction between Mismatch × Region × Group, suggesting that ERP effects may be topographically differentially distributed across older and younger adults.

**Table 2:** Statistical outputs from the overall omnibus repeated measures ANOVAs performed on the ERPs data with condition × mismatch × group × region design, *p* values < .05 are bolded, η_p_^2^ represents partial eta-squared effect sizes.

	df	300–500 ms			500–700 ms			700–900 ms		
		*F*	*p*	η_p_^2^	*F*	*p*	η_p_^2^	*F*	*p*	η_p_^2^
**Main-effects**										
Group	1,31	**15.12**	** < 0.001**	**0.32**	2.72	0.11	0.08	1.70	0.20	0.05
Condition	1,31	**49.47**	** < 0.001**	**0.62**	**9.53**	**0.005**	**0.23**	2.75	0.10	0.08
Mismatch	1,31	0.11	0.73	0.004	**29.29**	** < 0.001**	**0.48**	**41.23**	** < 0.001**	**0.57**
Region	14,434	**15.19**	** < 0.001**	**0.33**	**8.28**	** < 0.001**	**0.21**	**4.69**	**0.005**	**0.13**
**Two-way interactions**										
Condition × group	1,34	1.62	0.21	0.05	0.21	0.64	0.001	2.71	0.11	0.08
Mismatch × group	1,34	0.29	0.59	0.009	2.40	0.13	0.07	0.90	0.34	0.03
Region × group	14,434	**2.93**	**0.04**	**0.08**	0.73	0.49	0.02	0.38	0.73	0.01
Condition × mismatch	1,34	0.09	0.76	0.003	0.21	0.65	0.007	1.81	0.18	0.05
Condition × region	14,434	**5.99**	**0.002**	**0.16**	**5.79**	**0.002**	**0.15**	**12.52**	** < 0.001**	**0.28**
Mismatch × region	14,434	**2.09**	**0.01**	**0.06**	**5.05**	**0.002**	**0.14**	**6.55**	**0.001**	**0.17**
**Three-way interactions**										
Condition × mismatch × group	1,34	0.14	0.71	0.005	0.23	0.63	0.008	0.33	0.57	0.01
Condition × region × group	14,434	0.68	0.53	0.02	0.80	0.48	0.02	**2.97**	**0.02**	**0.09**
Mismatch × region × group	14,434	1.24	0.30	0.03	**3.22**	**0.02**	**0.09**	1.28	0.28	0.04
Condition × mismatch × region	14,434	1.94	0.12	0.06	1.91	0.10	0.05	0.68	0.60	0.02
**Four-way interaction**										
Condition × mismatch × region × group	14,434	1.55	0.20	0.04	0.87	0.49	0.02	0.63	0.63	0.02

Between 700 and 900 ms, responses to the mismatch sentences were expectedly more positive than sentences without a mismatch (2.05 μV vs. 0.72 μV). However, neither the main-effect of Group nor other critical interactions between Group and Mismatch reached statistical significance, signalling that those condition differences in grand-averaged ERPs were not modulated by age-effects in this late time window. Given the significant interactions between Group and Condition/Mismatch and Region in the 500–700 ms and 700–900 ms time-windows, we further analysed the ERP data for anaphoric and cataphoric conditions separately. The statistical outputs from subsequent ANOVAs computed with anaphor and cataphor conditions are displayed in Table 3; and mean amplitudes of difference waveform for particular group differences per ROI are given in Table 4

**Table 3:** Statistical outputs from subsequent rmANOVA models computed with anaphor and cataphor conditions separately.

	df	Anaphor sentences									Cataphor sentences								
		300–500 ms			500–700 ms			700–900 ms			300–500 ms			500–700 ms			700–900 ms		
		*F*	*p*	η_p_^2^	*F*	*p*	η_p_^2^	*F*	*p*	η_p_^2^	*F*	*p*	η_p_^2^	*F*	*p*	η_p_^2^	*F*	*p*	η_p_^2^
**Main-effects**																			
G	1,31	**5.96**	**0.02**	**0.16**	2.69	0.11	0.08	3.80	0.06	0.10	**16.40**	** < 0.001**	**0.34**	1.84	0.18	0.05	0.05	0.82	0.002
M	1,31	0.15	0.69	0.005	**14.35**	**0.001**	**0.31**	**11.65**	**0.002**	**0.27**	0.002	0.97	0.001	**15.19**	**0.001**	**0.32**	**20.63**	** < 0.001**	**0.40**
R	14,434	**9.39**	** < 0.001**	**0.24**	**6.18**	**0.003**	**0.16**	**3.42**	**0.03**	**0.10**	**14.86**	** < 0.001**	**0.32**	**8.82**	**0.001**	**0.22**	**9.91**	** < 0.001**	**0.24**
**Two-way interactions**																			
M × G	1,31	0.32	0.57	0.01	2.42	0.13	0.07	1.43	0.24	0.04	0.02	0.87	0.001	0.50	0.48	0.01	0.01	0.89	0.001
R × G	14,434	1.95	0.13	0.05	1.32	0.27	0.04	1.25	0.29	0.03	2.96	0.06	0.07	0.32	0.74	0.01	0.52	0.69	0.02
M × R	14,434	**3.15**	**0.03**	**0.09**	**5.62**	**0.002**	**0.15**	**4.12**	**0.01**	**0.11**	0.70	0.55	0.02	1.87	0.11	0.06	**4.60**	**0.002**	**0.12**
**Three-way interactions**																			
M × R × G	14,434	2.24	0.10	0.06	**3.21**	**0.03**	**0.09**	0.72	0.50	0.02	0.39	0.78	0.01	1.27	0.28	0.04	1.36	0.25	0.04

*p* values < 0.05 are bolded.

df,  degrees of freedom, η_p_^2 =^ partial eta squared, G,  Group, M,   Mismatch, R,   Region of Interest.

**Table 4:** Mean amplitude of difference waveform in microvolts (i.e. mismatch minus match; standard deviation is given in parenthesis) *in 500–700 ms time window *over the frontal regions, and statistical outputs from post-hoc group comparisons of computed per region of interest (ROI) and per condition.

ROIs	Anaphor sentences				Cataphor sentences			
	Young (SD)	Older (SD)	t (*p*) value	95%CIs	Young	Older	t (*p*) value	95%CIs
FL	**1.08 (1.83)**	− **0.95 (1.83)**	− **3.17 (0.003)**	**[**− **3.34, **− **0.72]**	1.66 (2.07)	0.46 (2.10)	− 1.63 (0.11)	[− 2.68, 0.29]
FM	1.25 (2.54)	− 0.44 (2.81)	− 1.82 (0.07)	[− 3.60, 0.20]	1.40 (2.98)	0.42 (2.19)	− 1.06 (0.29)	[− 2.88, 0.90]
FR	**1.46 (2.41)**	− **0.45 (2.27)**	− **2.33 (0.02)**	**[**− **3.59, **− **0.24]**	1.45 (2.13)	0.82 (2.15)	− 0.83 (0.41)	[− 2.15, 0.90]
FCL	**1.40 (1.77)**	− **0.18 (1.72)**	− **2.58 (0.01)**	**[**− **2.83, **− **0.33]**	1.67 (2.07)	0.67 (2.61)	− 1.23 (0.22)	[− 2.67, 0.66]
FCR	**1.61 (1.67)**	− **0.84 (2.33)**	− **2.43 (0.02)**	**[**− **3.12, **− **0.27]**	1.37 (1.82)	0.71 (2.38)	− 0.89 (0.37)	[− 2.15, 0.88]

FL,  frontal left; FM,  frontal midline; FR,  frontal right; FCL,  fronto-central left; FCR,  fronto-central right. CIs represent 95% confidence intervals.

*p* values < 0.05 are bolded.

### Anaphoric pronouns

Figure 1 displays plotted ERPs evoked by the anaphoric pronoun conditions. We observed a clear central/posterior positivity (i.e., P600) evoked in response to anaphoric mismatches in relevance to the match condition. The timing of the P600 component for the anaphoric pronoun conditions was similar across both groups; for instance, in the posterior regions, onset latency was around 300 ms for the older adults while it was 370 ms for the younger adults. However, the effect was more sustained in the younger group extending beyond 1000 ms, which was not the case in the older adults (see Fig. 1). Additionally, the older adults’ processing of anaphoric pronouns was characterised by the presence of a frontal negativity that lacked in the younger adults.

### 300–500 ms time-window

The older adults’ mean amplitude responses were more positive than the younger adults (2.92 μV vs. 1.46 μV), however, there were no significant main-effects of Mismatch nor interactions with Mismatch and Group in this time-window.

### 500–700 ms time-window

**Figure 2:**
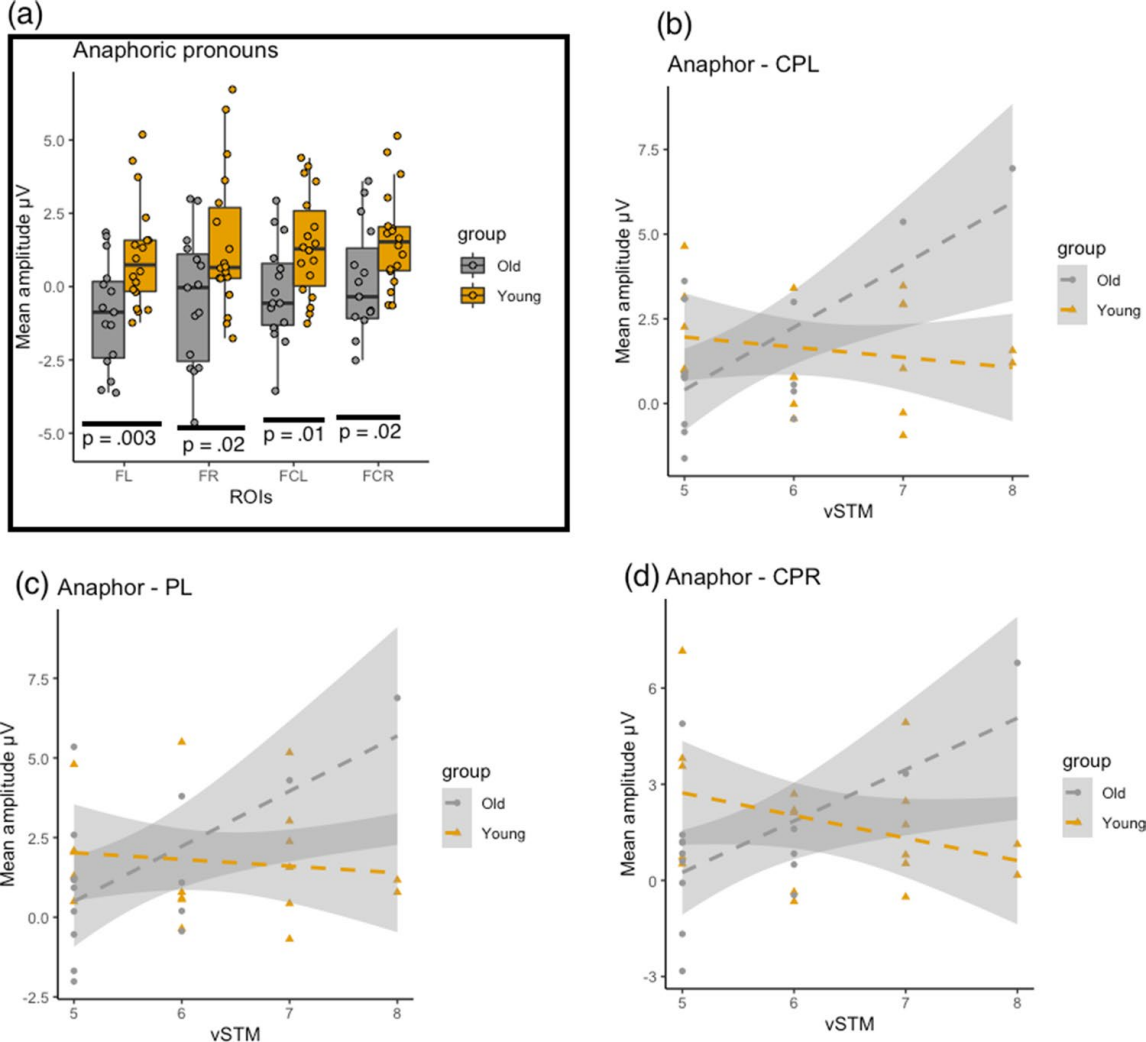
(**a**) Boxplots with mean ERP waveforms (mismatch minus match) in 500–700 ms time-window for anaphoric pronoun conditions plotted over frontal regions of interest showing frontal negativity in older adults as compared to younger adults (FL = frontal left, RF = Frontal Right, FCL = Fronto-Central Left, and FCR = Fronto-Central Right). Plotted regression lines for (**b**) Central-parietal Left (CPL), (**c**) Parietal Left (PL), and (**d**) Central-parietal Right (CPR) regions indicating associations between verbal short-term memory (vSTM) and mean amplitude of ERPs in 500–700 ms time-window. The plots were drawn using R version 3.6.0.

Anaphoric pronoun mismatches evoked more positive ERP effects in this time window as compared to their match counterparts (2.60 μV vs. 1.49 μV). Critically, there was a significant three-way interaction between Mismatch × Region × Group, without a significant main-effect of Group or any other interaction effects between Group/Mismatch and Group. The three-way interaction reflects an anterior negativity that emerged in the older adults’ processing of anaphoric mismatches. The older adults’ mean ERP amplitudes showed a negative-going waveform over the frontal regions, specially FL, FR, FCL, and FCR, as compared to the younger adults (see Table 4 and Fig. 2). No further significant group differences were observed in this time-window. Critically, the observed P600 effect evoked by the anaphoric pronoun conditions showed important associations with the older participants’ vSTM scores. These associations, evidenced with linear regression models, were particularly strong over the PL (*ß* = 1.51, SE = 0.55, *t* = 2.73, *p* = 0.017), CPL (*ß* = 1.84, SE = 0.52, *t* = 3.50, *p* = 0.003), and CPR (*ß* = 1.60, SE = 0.57, *t* = 2.79, *p* = 0.015). This suggests that older adults with higher span scores tended to have more positive amplitudes in ERP effects over these selected ROIs than those with relatively lower span scores. No significant modulation of the vSTM scores on the P600 amplitudes was observed in the younger adults, however. This is visually depicted in Fig. 2.

### 700–900 ms time-window

The ERP effects between 700 and 900 ms mirrored the effects in the 500–700 ms time-window. Specifically, the mismatch condition evoked greater positive responses than its match counterpart (2.10 μV vs. 1.10 μV). However, the absence of significant main-effect or interaction effects between Group and Mismatch allows us to suggest that the older adults’ brain potentials mimicked those of the younger adults during their processing of anaphoric pronouns in the late time window.

### Cataphoric pronouns

**Figure 3:**
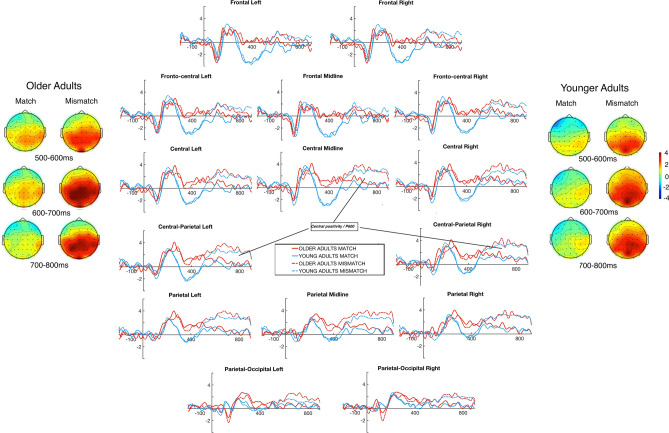
Grand averaged ERPs for cataphoric pronoun match and mismatch conditions across fifteen regions of interest, central positivity is indicated in the line plots. Dashed red lines represent mismatch conditions and solid lines represent match conditions. Topographic maps show distribution of averaged ERPs between 500 and 800 ms. Positive μV potential values are plotted up in the x-axes and time in millisecond is plotted in the y-axes. The plots were generated using the EEGLAB software toolbox.

Figure 3 displays plotted ERPs evoked by the cataphoric pronoun conditions, which showed central/posterior positivity (i.e., P600), without any frontal negativity effects, in both groups. The onset latencies of this positivity, which was strong over posterior and central-posterior ROIs, were similar in both groups with an onset at about 480 ms for the young and 450 ms for the older adults.

### 300–500 ms time-window

Evoked potentials in this time-window were characterised by the younger adults’ larger negative amplitudes compared to the older adults (− 1.24 μV vs. 1.04 μV). However, given the absence of significant effects of Mismatch, these negative-going waveforms cannot be affiliated with cataphoric pronoun-antecedent mismatches.

### 500–700 ms time-window

In this time-window, we found significant main-effects of Mismatch and Region. Expectedly, the mismatch cataphor condition evoked more positivity than its match counterpart (1.99 μV vs. 0.65 μV). However, since there were no interactions between Group and Mismatch, and no direct comparisons in any ROI reached statistical significance (see Table 4), one can safely assume that the observed P600 component was similar in both groups with regard to its amplitude and distribution. Furthermore, the positive amplitudes in the cataphoric pronoun conditions did not show any relationship with our participants’ vSTM scores (all *p*s > 0.46).

### 700–900 ms time-window

The ERP effects in this time-window were similar to those in the previous one; namely, there were significant main-effects of Mismatch and Region and not of Group. The mismatch cataphor condition evoked more positive responses on average than the match cataphor condition (1.99 μV vs. 0.32 μV). No significant effects of Group, however, indicate that the young and older adults’ electrophysiological responses were similar in this time-window.

## Discussion

We investigated age-related changes in anaphoric and cataphoric pronoun processing in adult French speakers and the affiliated temporal electrophysiological activity. The specific aims of the current study were (1) to uncover the extent to which processing of anaphoric and cataphoric pronouns is maintained in older adults as compared to younger adults, regarding behavioural and per-millisecond electrophysiological time-course of this processing; (2) to understand whether there are any processing differences for anaphoric and cataphoric pronouns in terms of younger and older adults’ brain responses.

The behavioural results showed that the older adults’ end-of-sentence responses were not less accurate than the younger adults. In fact, they even performed slightly better in responding to the cataphoric pronoun conditions than the younger adults, while there was no difference in the anaphoric pronoun conditions. In both groups, there were individual differences modulated by vSTM scores: the participants with a lower verbal memory span performed more poorly in detecting mismatches than those with a higher span. Regarding the participants’ response latencies, the older adults were not slower than the younger adults in detecting mismatches. For the ERP results, we observed a sustained central/parietally distributed positive component (P600) with an onset-latency between 300 and 500 ms in both the anaphor and cataphor conditions. Anaphoric pronoun-antecedent mismatches evoked more positive responses on average than their cataphoric counterparts. We found no direct age effects on distribution or timing of the P600 component in either condition pairs, although positive-going waves in the anaphor mismatch condition were rather more sustained in the younger adults extending beyond 900 ms. Interestingly, however, the older adults showed an anterior negativity, emerging over the frontal and fronto-central ROIs, during their processing of anaphoric conditions, which was absent in the younger adults. No such component emerged in the cataphoric conditions. The positive ERP amplitudes recorded over parietal and central-parietal regions in response to anaphoric pronoun mismatches were predicted by vSTM scores in the older adults but no such association emerged in the younger adults, suggesting that the older adults with higher verbal span, but not the younger ones, evoked more positive amplitudes.

Remarkably, the lack of group differences in positive amplitudes over critical ROIs was stable in both the anaphor and cataphor conditions, in contrast to a number of studies that report reduced and delayed potentials in typically ageing adults (see e.g.^45^). Our findings are fully reconcilable with Kemmer et al.^47^, who reported similar P600 effects for English-speaking groups of older and younger adults’ responses to gender-mismatches in reflexive pronouns. However, unlike Kemmer and colleagues, we found an additional anterior negativity in the older adults’ processing of anaphoric pronouns, which was not present either in the younger adults or in the cataphoric conditions. It is in fact not uncommon that pronoun resolution studies report the presence of an anterior negativity, which is thought to occur during resolving ambiguity that is associated with unknown antecedents or memory retrieval processes^32,40,51–53^. One possible explanation for the presence of an anterior negativity in the older adults only is greater demands for memory resources during anaphora processing. This is also supported by the fact that older adults tested in this study proved to have reduced verbal STM spans, as measured with a digit span task (see Method), compared to the younger adults. It is therefore conceivable that the older adults’ anaphoric pronoun comprehension is affiliated with an increased processing demand.

The answer to the question “*Does syntactic processing decline with age?*” is not a straightforward one. Following the syntactic decline accounts and studies that showed reduced pronoun processing capability in ageing adults^5,7,43^, we would expect the older adults to perform more poorly in their responses to the end-of-sentence acceptability judgement task. Contrary to expectations, we found that the older adults performed similarly to the younger adults (and in fact slightly more accurately with the cataphoric pronouns), consistent with studies conducted by Tyler and colleagues^16,19^. Therefore, the data from the current study seem to support the view that compensatory functional recruitment of additional neural regions renders syntactic processing resilient in typically ageing adults. Although our study did not use functional imaging to localise which precise regions are additionally recruited, the time-course of electrophysiological potentials suggests that anaphoric-pronoun processing, but not cataphoric-pronoun processing, required increased processing demands with rather more positive P600 amplitudes in both groups and with an additional anterior negativity in the older adults. The anterior negativity may therefore point to additional brain activity compensating for these increased processing demands in the older adults. Furthermore, increased processing demands for anaphoric-pronoun processing, which yielded more positivity in both groups as compared to cataphoric-pronoun processing, may be affiliated with retrieving the correct antecedent from memory from an established set of potential referents.

However, we caution the reader that these findings should not be construed as syntactic abilities being unitarily resilient in the healthy ageing, as there seems to be individual variation, particularly in association with verbal memory capacity^7,10,54^. We found that the P600 effect evoked by the anaphoric mismatch condition was modulated by vSTM capacity in the older adults, which suggested that the amplitude of positive-going wave was rather reduced in the older adults with considerably lower verbal storage capacity, see also^48,55^ for discussions on modulation of P600 amplitude by memory constraints during syntactic processing. Importantly, vSTM scores also modulated both the older and younger groups’ behavioural accuracy data, albeit not the response times. This brings us to the issue of whether or not potential age effects on syntactic-processing ability are prone to interpretive and post-interpretive distinctions. According to Waters and Caplan’s model^24^, sentence interpretation ability is sensitive to ageing effects during offline comprehension (i.e., post-interpretive), but not online, see Kemper et al.^56^ for discussion. A possible explanation for post-interpretive difficulty in older adults is explained by an account that predicts that offline judgements require different and/or additional cognitive processes from per-millisecond time course of online sentence comprehension^23,49,50^. However, the existing evidence is mixed, see for instance, Alatorre-Cruz et al.^48^ who found no critical age differences in older adults’ response accuracy in a sentence interpretation task with high WM-load sentences. The data from our study resemble these results: WM-intensive sentence interpretation ability is age-sensitive (see also e.g.^7,12,13,15,48^), and the older adults’ syntactic processing ability as measured by their behavioural responses is not necessarily reduced, suggesting that our older participants did not encounter post-interpretative (i.e., relating to offline judgements) difficulties to a large extent. A coherent line of conclusion we reach at this point, as also pointed out by an anonymous reviewer, is that the WM resources seem to be affected for holding a filler (i.e., a pronoun) in memory while searching for the correct antecedent, but when the antecedent is found, the post-interpretive processes, which apply when making an acceptability judgement, remain unaffected. Nonetheless, in the absence of data detailing the older adults’ domain-general cognitive abilities in addition to memory, we cannot further contemplate on how these post-interpretive processes are maintained.

We further attempted to uncover possible differences between older and younger adults processing of anaphoric and cataphoric pronouns. According to a view, processing anaphoric and cataphoric pronouns are assumed to rely on different cognitive processes, that is, while anaphoric pronoun resolution requires storing and retrieval of antecedents, cataphoric resolution is thought to rely on an antecedent-search mechanism^28,29,41,42^. Research has shown that the P600 component is consistently reported in studies using gender-mismatch pronoun processing comprehension tasks^27,30–33^, and is known to be associated with sentence repair processes^35–38^. Our results are compatible with this view, as the current study found a P600 effect for both anaphoric and cataphoric pronoun conditions, but there were critical differences between the two conditions. The cataphoric pronoun conditions evoked reduced amplitudes of positivity and longer response times at the end-of-sentence judgement task compared to their anaphoric counterparts. Regarding cataphoric pronouns, Pablos et al.^29^ reported an anterior negativity in constrained dependencies but no ERP effects for unconstrained cataphoric dependencies. The authors argued that the presence of anterior negativity in response to gender-mismatch in cataphoric pronouns might be affiliated with a failure to find appropriate antecedents, which prevails the effects of gender-mismatch. Our findings are however at odds with the claims that gender-mismatch effects are overridden in cataphoric pronoun processing, as we found that gender-mismatch in cataphoric pronouns evoked a clear P600 effect similar to that in anaphoric pronoun dependencies. It is indispensable to note that Pablos et al.^29^ used constrained pronouns (i.e., where the antecedent cannot structurally bind the pronoun) and hence, the observed anterior negativity perhaps results from these linguistic constraints, as they discussed. Without a doubt, our study provides support that gender-mismatch in cataphoric pronouns elicits a P600 effect time-locked to the antecedent region, similar to anaphoric pronouns but with reduced P600 amplitude. This is reconcilable with Kennison et al.^41^ who reported an asymmetry between anaphoric and cataphoric pronoun resolution in word-by-word reading data, despite similar accuracy rates, and argued that readers interpret co-reference relations similarly.

## Conclusions

This study provides critical outcomes regarding sentence processing ability and its electrophysiological time course in healthy ageing adults as compared to younger adults. Our findings suggest that behavioural and electrophysiological brain responses when processing anaphoric/cataphoric pronouns are not compromised in older adults compared to younger ones. However, for anaphoric pronouns (i.e., when the pronoun comes after its antecedent) an additional anterior negativity is found in older adults but not in younger ones. We interpret the presence of this anterior negativity as evidence for functional recruitment of additional brain activity compensating for declining memory resources in older adults. This additional recruitment in older adults would then assist in antecedent storage and retrieval (i.e., anaphor interpretation), but not in antecedent probing (i.e., cataphor search mechanisms). Consequently, an important line of conclusion we suggest is that, whereas pronoun processing in older adults is not necessarily compromised in any measurable way, there are important individual differences linked with verbal memory constraints.

## Method

### Participants

A total of 33 participants were recruited for the ERP study, these included two groups of young (n = 18, 11 females, mean age = 22.05, SD = 3.88, ranges = 19–35) and older (n = 15, 11 females, mean age = 65.73, SD = 9.0, ranges = 57–88) native French speaking and community-dwelling individuals. All participants were right-handed as measured by the Edinburg Handedness Inventory^57^ (each participant scored > 70% right-handed). Table 5 displays further demographic and cognitive measures on our participants, none of whom showed any potential cognitive impairments, as they scored above a confident score (> 23) on the French version of the Mini Mental State Examination (MMSE)^58^. All the participants had normal or corrected to normal vision and none reported any significant impairment or drug use that may affect their language processing ability. The study was approved by the Ethics Commission of the Université Côte d’Azur (CERNI-File No. 2019-2) and followed the principles of the Declaration of Helsinki. All participants provided written informed consent. Please note that initially 37 participants were recruited for the study, but during pre-processing, 2 older and 2 younger individuals were removed due to extensive artefacts.

**Table 5:** Demographic and cognitive details of young and older groups of participants in the ERP experiment (MMSE = Mini Mental State Examination, vSTM = verbal short-term memory, nvSTM = non-verbal short-term memory, PE = print exposure, CIs represent 95% confidence intervals).

	Means (SD)		Test statistics	[95% CIs]
	Young (n = 18)	Older (n = 15)		
MMSE	29.44 (0.51)	28.26 (2.05)	*t*(15.45) = − 2.16, *p* = 0.004	[− 2.33, − 0.02]
vSTM	6.27 (1.01)	5.46 (0.99)	*t*(30.21) = − 2.31, *p* = 0.027	[− 1.52, − 0.09]
nvSTM	3.83 (1.15)	3.40 (0.98)	*t*(30.96) = − 1.16, *p* = 0.252	[− 1.19, 0.32]
PE	5.50 (4.36)	5.93 (5.82)	*t*(25.57) = 0.23, *p* = 0.814	[− 3.31, 4.18]

In order to detail the participants’ cognitive profiles, verbal short term memory (vSTM) skills were tested with the forward digit span task from the Wechsler Adult Intelligence Scale-III^59^. Non-verbal STM (nvSTM) was measured using the Corsi block-tapping test^60^. The nvSTM task required forward recall canonically increasing sequences of finger taps from two to nine blocks per span length. Print exposure was tested using the Author Recognition Task^61^ in which the participants were given a checklist of 130 (65 real and 65 fake) author names and were asked to check the author names that they recognized. For each participant, a score of print exposure (PE) was calculated by subtracting the false from the accurate responses. Results from a set of Welch t-tests conducted on the cognitive measures showed that the older group performed less well on vSTM but not on nvSTM or Print Exposure tasks (see Table 5).

### Materials

A total of 52 sentences were created with four conditions: Anaphor match/mismatch, (1), and Cataphor match/mismatch, see (2), summing up to a total of 208 sentences. Across the four conditions, we manipulated gender agreement between the personal pronoun (*il/elle* ‘he/she’) and the antecedent (a proper name). Genders of pronouns and proper names were counterbalanced in the experimental design, and hence, half of the sentences contained female names (n = 26). Efforts were given to select proper names that are stereotypically either female or male in French (e.g., *Tania* or *Jules*) in order to focus on syntactic gender and avoid potential biological gender effects. The sentence materials used in the experiments were tediously evaluated using a series of pre-experimental questionnaire studies on groups of French native speakers to examine (1) gender stereotypes of proper names, (2) any potential contextual bias in pronouns, and (3) cloze probability of critical word segments (for a detailed description and outcomes from these questionnaire studies, see supplementary information online).

**Table:** 

(1) Anaphor match/mismatch											
Jules	persuade	des	amis	de	parler	parce qu'	il/elle*	est	vraiment	très	aphone
Jules	persuades	some	friends	to	talk	because	he/she	is	really	very	voiceless
‘Jules persuades some friends to talk because he/she is quite voiceless.’											

**Table:** 

(2) Cataphor match/mismatch											
Parce qu'	il/*elle	est	vraiment	très	aphone	Jules	persuade	des	amis	de	parler
Because	he/she	is	really	very	voiceless	Jules	persuades	some	friends	to	talk
‘Because he/she is quite voiceless Jules persuades some friends to talk.’											

### Procedures

The sentence stimuli were programmed within a word-by-word moving-window paradigm and presented using the E-Prime 3.0 software (Psychology Software Tools, Pittsburgh, PA)^62^. The participants were seated in front of a presentation monitor within a comfortable reading distance located in a soundproof booth with electromagnetic-shielding. The participants were instructed to read the sentences silently, and at the end of each sentence, to respond to an end-of-sentence acceptability judgement task by pressing (j = ‘yes’) and (f = ‘no’) keyboard buttons. The judgement question asked the participants *Cette phrase est-elle acceptable*? (‘Is this sentence acceptable?’), and stayed until the participants provided a response. The sentence stimuli were presented visually word-by-word, each word appeared in the centre of the screen in Courier New font (32 pts.) for 500 ms followed by 350 ms blank screen. The experiment started with a practice session including four sentences to make sure the participants understood the task correctly. Following a standard Latin-square design, the experimental sentences were distributed over two lists, and the match and mismatch conditions were counterbalanced across the two lists, therefore, a participant saw 104 experimental sentences (i.e., 26 experimental items per participant per condition). The experimental sentences in each list were programmed in eight blocks, in each of which the number of sentences from different conditions were equal. Anaphor and cataphor condition manipulations of the same sentence were given in different blocks distant from each other. The participants only saw either a match or mismatch version of the same sentence material. Breaks were planned between each block in an experiment. The whole experiment took about 50 to 70 min in total (about 2 h including EEG preparation). The participants received 20 Euros as compensation for their time.

### EEG acquisition and pre-processing

The EEG signals were recorded via a 64-channel (standard 10–20 system) elastic Quick-Cap (Compumedics Inc.). An additional electrode integrated within the cap (in proximity to Fz) was used as the ground electrode. Horizontal and vertical EOG signals were recorded through bipolar electrodes, and additional two electrodes placed on the mastoids to be used as offline reference were integrated within the cap. The signals were digitally amplified, using the Neuroscan 64-channel EEG system amplifier, at the sampling rate of 1000 Hz (i.e., one data point per millisecond). During recording, impedance in all electrode sites was below 10 kΩ.

The EEG signals were processed using EEGLAB software toolbox^63^ in MATLAB version R2019b. The signals were down-sampled to 256 Hz for computational purposes, and re-referenced to the mastoid electrodes. We applied low-pass and high-pass at the cut-off frequencies of 0.1–46 Hz. ERPs were segmented for a 2000 ms time frame (400 before and 1600 ms after the later pronoun/referent was presented). In each segmented trial, average data were corrected to − 400 ms baseline. Trials with excessive artefacts exceeding 100 μV were semi-automatically detected and rejected. Total amount of rejected trials corresponded to 8.4% in the young group and 9.2% in the older group. Data rejection was not greater than 20% of all trials per condition per subject. Independent Component Analysis (ICA) was performed using the Picard algorithm^64^. Components pertaining to ocular, muscular artefacts and channel line noise were automatically labelled with the ICLabel classifier^65^, and were removed before further analyses.

### Data analysis

For behavioural data, response latencies were analysed using linear mixed-effects regression models, and accuracy of responses were analysed using generalized mixed-effects logistic regression in R version 3.6.0^66^. Categorical fixed factors including Group (Older vs. Younger), Condition (Anaphor vs. Cataphor) and Mismatch, were re-centred using sum-coding (i.e. − 0.5 vs. 0.5 rather than 1–0 binary dummy coding) to avoid potential biases in regression. Response times were log transformed prior to the analyses, subjects and items were added as random intercept (and slopes where applicable)^67^. Results from EEG analysis were averaged across items (in −/ + μV) and were extracted for each participant per condition. The electrode sites were clustered into 15 regions of interest (ROI): frontal left (F1, F3, F5), frontal right (F2, F4, F6), fronto-central left (FC1, FC2, FC3), frontal midline (FPz, Fz), fronto-central right (FC2, FC4, FC6), central left (C1, C3, C5), central midline (FCz, Cz, CPz), central right (C2, C4, C6), central-parietal left (CP1, CP3, CP5) central-parietal right (CP2, CP4, CP6), parietal left (P1, P3, P5), parietal midline (Pz, POz, Oz), parietal right (P2, P4, P6), parietal-occipital left (PO1, PO3, O1), and parietal-occipital right (PO1, PO3, O1). Following an initial visual inspection of ERP effects (see Luck^68^) mean amplitudes per ROI were analysed across three time-windows (300–500 ms, 500–700 ms and 700–900 ms) using separate repeated measures ANOVAs with a 2 × 2 × 2 × 15 design including the factors of Condition (Anaphor vs. Cataphor) × Mismatch (Match vs. Mismatch) × Group (Older vs. Younger) × Region (15 ROIs as described above). Subsequent ANOVA models were then built for the anaphor and cataphor conditions separately with Mismatch × Group × Region design to further inspect group differences. The statistical significance was *p* < 0.05, and *p*-values were reported after the Geisser-Greenhouse correction. A complementary set of linear regression models was built to examine whether and how our participants’ verbal memory span (i.e. vSTM) modulated their P600 amplitudes. The P600 amplitudes per participant were calculated as the voltage amplitude of the difference for mismatch conditions minus the baseline (i.e., match conditions).

## Supplementary information

Supplementary Information

## Data Availability

The datasets generated and analysed during the current study are available from the corresponding author on reasonable request.

## References

[1] Kemper S, Liu C-J (2007). Eye movements of young and older adults during reading. Psychol. Aging.

[2] Poulisse C, Wheeldon L, Segaert K (2019). Evidence against preserved syntactic comprehension in healthy aging. J. Exp. Psychol. Learn. Mem. Cognit..

[3] Stine-Morrow EA, Shake MC, Miles JR, Noh SR (2006). Adult age differences in the effects of goals on self-regulated sentence processing. Psychol. Aging.

[4] Wingfield A, Peelle JE, Grossman M (2003). Speech rate and syntactic complexity as multiplicative factors in speech comprehension by young and older adults. Aging Neuropsychol. Cognit..

[5] Light LL, Capps JL (1986). Comprehension of pronouns in young and older adults. Dev. Psychol..

[6] Obler LK, Fein D, Nicholas M, Albert ML (1991). Auditory comprehension and aging: Decline in syntactic processing. Appl. Psycholinguist..

[7] Payne BR (2014). Aging and individual differences in binding during sentence understanding: evidence from temporary and global syntactic attachment ambiguities. Cognition.

[8] Hasher L, Zacks RT (1988). Psychology of Learning and Motivation, 22.

[9] Salthouse TA (1996). The processing-speed theory of adult age differences in cognition. Psychol. Rev..

[10] Kemtes KA, Kemper S (1997). Younger and older adults' on-line processing of syntactically ambiguous sentences. Psychol. Aging.

[11] Daneman M, Carpenter PA (1980). Individual differences in working memory and reading. J. Verb. Learn. Verb. Behav..

[12] Angwin AJ (2006). Searching for the trace: The influence of age, lexical activation and working memory on sentence processing. J. Psycholinguist. Res..

[13] Caplan D, DeDe G, Waters G, Michaud J, Tripodis Y (2011). Effects of age, speed of processing, and working memory on comprehension of sentences with relative clauses. Psychol. Aging.

[14] Stine-Morrow EAL, Ryan S, Leonard S (2000). Age differences in on-line syntactic processing. Exp. Aging Res..

[15] Kemper S, Greiner LH, Marquis JG, Prenovost K, Mitzner TL (2001). Language decline across the life span: findings from the nun study. Psychol. Aging.

[16] Tyler LK (2009). Preserving syntactic processing across the adult life span: the modulation of the frontotemporal language system in the context of age-related atrophy. Cereb. Cortex.

[17] Meunier D, Stamatakis EA, Tyler LK (2014). Age-related functional reorganization, structural changes, and preserved cognition. Neurobiol. Aging.

[18] Shafto MA, Tyler LK (2014). Language in the aging brain: the network dynamics of cognitive decline and preservation. Science.

[19] Campbell KL (2016). Robust resilience of the frontotemporal syntax system to aging. J. Neurosci..

[20] Samu D (2017). Preserved cognitive functions with age are determined by domain-dependent shifts in network responsivity. Nat. Commun..

[21] Beese C, Meyer L, Vassileiou B, Friederici AD (2017). Temporally and spatially distinct theta oscillations dissociate a language-specific from a domain-general processing mechanism across the age trajectory. Sci. Rep..

[22] Peelle JE, Troiani V, Wingfield A, Grossman M (2009). Neural processing during older adults’ comprehension of spoken sentences: age differences in resource allocation and connectivity. Cereb. Cortex.

[23] Wingfield A, Grossman M (2006). Language and the aging brain: patterns of neural compensation revealed by functional brain imaging. J. Neurophysiol..

[24] Waters GS, Caplan D (2001). Age, working memory, and on-line syntactic processing in sentence comprehension. Psychol. Aging.

[25] Badecker W, Straub K (2002). The processing role of structural constraints on interpretation of pronouns and anaphors. J. Exp. Psychol. Learn. Mem. Cogn..

[26] Chow W-Y, Lewis S, Phillips C (2014). Immediate sensitivity to structural constraints in pronoun resolution. Front. Psychol..

[27] Harris T, Wexler K, Holcomb P (2000). An ERP investigation of binding and coreference. Brain Lang..

[28] Kazanina N, Lau EF, Lieberman M, Yoshida M, Phillips C (2007). The effect of syntactic constraints on the processing of backwards anaphora. J. Mem. Lang..

[29] Pablos L, Doetjes J, Ruijgrok B, Cheng LL-S (2015). Active search for antecedents in cataphoric pronoun resolution. Front. Psychol..

[30] Schmitt BM, Lamers M, Münte TF (2002). Electrophysiological estimates of biological and syntactic gender violation during pronoun processing. Cognit. Brain Res..

[31] Osterhout L, Bersick M, McLaughlin J (1997). Brain potentials reflect violations of gender stereotypes. Mem. Cognit..

[32] Osterhout L, Mobley LA (1995). Event-related brain potentials elicited by failure to agree. J. Mem. Lang..

[33] Silva-Pereyra J, Gutierrez-Sigut E, Carreiras M (2012). An ERP study of coreference in S panish: semantic and grammatical gender cues. Psychophysiology.

[34] Carreiras M, Garnham A, Oakhill J (1993). The use of superficial and meaning-based representations in interpreting pronouns: evidence from Spanish. Eur. J. Cognit. Psychol..

[35] Osterhout L, Holcomb PJ (1992). Event-related brain potentials elicited by syntactic anomaly. J. Mem. Lang..

[36] Hagoort P, Brown CM (1999). Gender electrified: ERP evidence on the syntactic nature of gender processing. J. Psycholinguist. Res..

[37] Kaan E, Swaab TY (2003). Repair, revision, and complexity in syntactic analysis: an electrophysiological differentiation. J. Cognit. Neurosci..

[38] Kaan E, Harris A, Gibson E, Holcomb P (2000). The P600 as an index of syntactic integration difficulty. Lang. Cognit. Process..

[39] Van Berkum JJ, Koornneef AW, Otten M, Nieuwland MS (2007). Establishing reference in language comprehension: an electrophysiological perspective. Brain Res..

[40] Nieuwland MS (2014). “Who’s he?” Event-related brain potentials and unbound pronouns. J. Mem. Lang..

[41] Kennison SM, Fernandez EC, Bowers JM (2009). Processing differences for anaphoric and cataphoric pronouns: implications for theories of discourse processing. Discourse Process..

[42] Van Gompel RP, Liversedge SP (2003). The influence of morphological information on cataphoric pronoun assignment. J. Exp. Psychol. Learn. Mem. Cogn..

[43] Leonard CL, Waters GS, Caplan D (1997). The influence of contextual information on the resolution of ambiguous pronouns by younger and older adults. Appl. Psycholinguist..

[44] Shake MC, Stine-Morrow EA (2011). Age differences in resolving anaphoric expressions during reading. Aging Neuropsychol. Cognit..

[45] Wlotko EW, Lee CL, Federmeier KD (2010). Language of the aging brain: event-related potential studies of comprehension in older adults. Lang. Linguist. Compass.

[46] Beese C, Vassileiou B, Friederici AD, Meyer L (2019). Age differences in encoding-related alpha power reflect sentence comprehension difficulties. Front. Aging Neurosci..

[47] Kemmer L, Coulson S, De Ochoa E, Kutas M (2004). Syntactic processing with aging: an event-related potential study. Psychophysiology.

[48] Alatorre-Cruz GC (2018). Effects of age and working memory load on syntactic processing: an event-related potential study. Front. Hum. Neurosci..

[49] Peelle JE, de Zubicaray GI, Schiller NO (2019). The Oxford Handbook of Neurolinguistics.

[50] Davis SW, Zhuang J, Wright P, Tyler LK (2014). Age-related sensitivity to task-related modulation of language-processing networks. Neuropsychologia.

[51] Karimi H, Swaab TY, Ferreira F (2018). Electrophysiological evidence for an independent effect of memory retrieval on referential processing. J. Mem. Lang..

[52] Fiorentino R, Covey L, Gabriele A (2018). Individual differences in the processing of referential dependencies: evidence from event-related potentials. Neurosci. Lett..

[53] Nieuwland MS, Van Berkum JJ (2006). Individual differences and contextual bias in pronoun resolution: Evidence from ERPs. Brain Res..

[54] DeDe G, Caplan D, Kemtes K, Waters G (2004). The relationship between age, verbal working memory, and language comprehension. Psychol. Aging.

[55] Vos SH, Gunter TC, Schriefers H, Friederici AD (2001). Syntactic parsing and working memory: the effects of syntactic complexity, reading span, and concurrent load. Lang. Cognit. Process..

[56] Kemper S, Crow A, Kemtes K (2004). Eye-fixation patterns of high-and low-span young and older adults: down the garden path and back again. Psychol. Aging.

[57] Oldfield RC (1971). The assessment and analysis of handedness: the Edinburgh inventory. Neuropsychologia.

[58] Folstein MF, Folstein SE, McHugh PR (1975). Mini-mental state. A practical method for grading the cognitive state of patients for the clinician. J. Psychiatr. Res..

[59] Wechsler D (2008). Wechsler Adult Intelligence Scale-Fourth Edition (WAIS–IV).

[60] Corsi, P. *Memory and the medial temporal region of the brain*. Unpublished doctoral dissertation, McGill University, Montreal, QB (1972).

[61] Acheson DJ, Wells JB, MacDonald MC (2008). New and updated tests of print exposure and reading abilities in college students. Behav. Res. Methods.

[62] Psychology Software Tools, I. *E-Prime 3.0* (2016). https://www.pstnet.com. Accessed 10 April 2019.

[63] Delorme A, Makeig S (2004). EEGLAB: an open source toolbox for analysis of single-trial EEG dynamics including independent component analysis. J. Neurosci. Methods.

[64] Ablin, P., Cardoso, J.-F. & Gramfort, A. In *2018 IEEE International Conference on Acoustics, Speech and Signal Processing (ICASSP)* 4464–4468 (IEEE).

[65] Pion-Tonachini L, Kreutz-Delgado K, Makeig S (2019). ICLabel: an automated electroencephalographic independent component classifier, dataset, and website. NeuroImage.

[66] R-Core-Team. R: A Language and Environment for Statistical Computing. Vienna, Austria. https://www.r-project.org/. Accessed 26 April 2019.

[67] Baayen RH, Davidson DJ, Bates D (2008). Mixed-effects modeling with crossed random effects for subjects and items. J. Mem. Lang..

[68] Luck SJ (2014). An Introduction to the Event-Related Potential Technique.

