# Supplementary material for: Electrophysiological differences in older and younger adults’ anaphoric but not cataphoric pronoun processing in the absence of age-related behavioural slowdown
Source: Sci Rep. 2020 Nov 6;10:19234. doi: 10.1038/s41598-020-75550-3 (PMC7648082; doi:10.1038/s41598-020-75550-3)
Supplement: Supplementary file 1 — Supplementary Information [file 41598_2020_75550_MOESM1_ESM.pdf]

S. Arslan et al.

Supplementary Information for "*Electrophysiological differences in older and younger adults' anaphoric but not cataphoric pronoun processing in the absence of age-related behavioural slowdown"*

Evaluation of the sentence material used in the experiments.

Gender stereotypes. We normed gender stereo-typicality of our proper names in an offline questionnaire using 10-point Likert scale (1 = very masculine, 10 = very feminine) with a total number of 277 native French speakers (*M*age=26.68, SD=10.20). Ratings to female and male names ranged between 9.92-8.60 and 1.17-1.61, respectively. Mean ratings between female and male names were significantly different [9.14 vs. 1.27; $t(14402) = -249.83$, $p < 0.001$].

Contextual bias. We further tested if there was any referential bias in our sentence material rendering interpretation of the pronouns relatively ambiguous. The sentences were programmed in an offline sentence judgement questionnaire in anaphor and cataphor conditions (mismatch conditions were not used). Twenty-two native French speakers were given this questionnaire (13 females, *M*age=26.17, SD=9.3), and were asked to answer a comprehension question for each sentence to identify the most appropriate referent (e.g., Question: *Qui est très aphone?* 'Who is very voiceless', Options: a. Jules, b. Another person). Results from the questionnaire showed that French native speakers considered the critical proper names (e.g., Jules/Tania) as potential antecedents for our critical pronouns in 94.5% of the time for the anaphor condition, and 92.6% for the cataphor condition. No significant differences were found between the two conditions ($t(1121.4) = -1.36$, $p = 0.18$, 95%CIs [-0.04, 0.009]).

Cloze probability. We designed a cloze probability task to find whether uses of third person pronouns at the critical word segment were expected. Twenty native French speakers (15 females, *M*age = 26; SD = 6.1) were given the above-described 52 experimental sentences with the critical segment of the sentence from the pronoun region onwards truncated in both anaphor and cataphor conditions (e.g., *Jules persuade des amis de parler parce* ___. ‘Jules persuaded friends to talk because ___’). The participants were asked to complete the reminder of the sentences. We counted uses of third person pronouns of interest (i.e., il/elle) in clear reference to sentence-initial proper names, proportioned to all words produced for this segment. The results revealed an appropriate personal pronoun was produced in 77% of the time in the anaphor condition and in 81% for the cataphor condition. No condition differences were found ($t(1085.8) = -1.47$, $p = 0.15$, 95% CIs [-0.08, 0.01]), suggesting that our critical pronouns are very much expected at the critical segment equally likely.
